# Mid-wall striae fibrosis predicts heart failure admission, composite heart failure events, and life-threatening arrhythmias in dilated cardiomyopathy

**DOI:** 10.1038/s41598-022-05790-y

**Published:** 2022-02-02

**Authors:** Yanish Purmah, Aidan Cornhill, Lucy Y. Lei, Steven Dykstra, Yoko Mikami, Alessandro Satriano, Dina Labib, Jacqueline Flewitt, Sandra Rivest, Rosa Sandonato, Michelle Seib, Andrew G. Howarth, Carmen P. Lydell, Bobak Heydari, Naeem Merchant, Michael Bristow, Louis Kolman, Nowell M. Fine, James A. White

**Affiliations:** 1 Stephenson Cardiac Imaging Centre, Libin Cardiovascular Institute of Alberta, University of Calgary, #0700, SSB, Foothills Medical Centre, 1403-29th St. NW, Calgary, AB T2N2T9 Canada; 2 Department of Diagnostic Imaging, Cumming School of Medicine, University of Calgary, Calgary, AB Canada; 3 Department of Cardiac Sciences, Cumming School of Medicine, University of Calgary, Calgary, AB Canada

**Keywords:** Cardiology, Cardiomyopathies

## Abstract

Heart failure (HF) admission is a dominant contributor to morbidity and healthcare costs in dilated cardiomyopathy (DCM). Mid-wall striae (MWS) fibrosis by late gadolinium enhancement (LGE) imaging has been associated with elevated arrhythmia risk. However, its capacity to predict HF-specific outcomes is poorly defined. We investigated its role to predict HF admission and relevant secondary outcomes in a large cohort of DCM patients. 719 patients referred for LGE MRI assessment of DCM were enrolled and followed for clinical events. Standardized image analyses and interpretations were conducted inclusive of coding the presence and patterns of fibrosis observed by LGE imaging. The primary clinical outcome was hospital admission for decompensated HF. Secondary heart failure and arrhythmic composite endpoints were also studied. Median age was 57 (IQR 47–65) years and median LVEF 40% (IQR 29–47%). Any fibrosis was observed in 228 patients (32%) with MWS fibrosis pattern present in 178 (25%). At a median follow up of 1044 days, 104 (15%) patients experienced the primary outcome, and 127 (18%) the secondary outcome. MWS was associated with a 2.14-fold risk of the primary outcome, 2.15-fold risk of the secondary HF outcome, and 2.23-fold risk of the secondary arrhythmic outcome. Multivariable analysis adjusting for all relevant covariates, inclusive of LVEF, showed patients with MWS fibrosis to experience a 1.65-fold increased risk (95% CI 1.11–2.47) of HF admission and 1-year event rate of 12% versus 7% without this phenotypic marker. Similar findings were observed for the secondary outcomes. Patients with LVEF > 35% plus MWS fibrosis experienced similar event rates to those with LVEF ≤ 35%. MWS fibrosis is a powerful and independent predictor of clinical outcomes in patients with DCM, identifying patients with LVEF > 35% who experience similar event rates to those with LVEF below this conventionally employed high-risk phenotype threshold.

## Introduction

Heart failure (HF) is a leading cause of morbidity and mortality, affecting approximately 2% of the adult population^1^. Idiopathic non-ischemic dilated cardiomyopathy (DCM) contributes significantly to this burden^2^; estimated to account for 8% of all HF patients^3^ and up to 19% of those with an left ventricular ejection fraction (LVEF) of < 35%^4^. Despite the practical adoption of LVEF-based thresholds in clinical trials for the stratification of high-risk DCM patients^5^, substantial evidence now supports a strong incremental role for myocardial fibrosis on late gadolinium enhancement (LGE) magnetic resonance imaging (MRI) to define high-risk phenotypes in this referral population^6–12^.

Mid-wall striae (MWS) fibrosis of the basal septum is observed in approximately one-third of DCM patients referred for LGE-MRI^13^. Across numerous studies this marker has been associated with elevated risk of all-cause death^6,7,10,14^ and arrhythmic death or appropriate ICD therapy^7–10,14–16^; these findings now confirmed in a multicentre setting^17^. However, the value of this unique marker to identify patients at elevated risk of HF hospitalization, an important contributor to patient morbidity and healthcare cost utilization, is poorly explored. Despite included within composite endpoints of several studies^6,8–10,12^, the independent utility of MWS fibrosis to identify patients at elevated incident risk of HF hospitalization has not been previously explored due to limited population size.

In a large, prospectively recruited cohort of patients with DCM undergoing LGE-MRI we assessed the independent prognostic utility of MWS fibrosis to predict incident HF admission. Two composite secondary outcomes were also investigated, these focussed on the impact of MWS fibrosis on broader HF-related outcomes (heart transplantation, left ventricular assist device (LVAD) implantation or death) and arrhythmia-related outcomes (appropriate ICD therapy, sudden cardiac death (SCD), survived sudden cardiac arrest (SCA), or sustained VT requiring cardioversion). All analyses were stratified for patients with an LVEF above versus below conventional high-risk phenotype thresholds.

## Methods

This was a prospective observational cohort study of subjects recruited between January 2015 and May 2018 and followed for a minimum of 12 months. The study was a sub-cohort analysis of the Cardiovascular Imaging Registry of Calgary (CIROC) at the Libin Cardiovascular Institute (NCT04367220). As previously described^18^, all patients in Southern Alberta referred for clinically indicated Cardiac MRI are prospectively enrolled with data collection performed using commercial software (cardioDI™, Cohesic Inc., Calgary) to deliver tablet-based patient engagement and consent, standardized patient-reported health questionnaires, indication-driven test protocolling and standardized reporting, followed by automated linkage to electronic health record (EHR) data.

Patients were considered eligible based on the following criteria: (1) referral for LGE-MRI for the evaluation of DCM; (2) confirmation of LVEF ≤ 50% by MRI-based quantification; (3) no prior clinical history of, or prior ICD-10 coded occurrence of myocardial infarction, percutaneous revascularization, or coronary artery bypass grafting (CABG); and (4) no LGE-based evidence of myocardial infarction, as defined by sub-endocardial pattern LGE in a typical coronary artery distribution. Patients with any clinically documented history of congenital heart disease, severe valvular insufficiency or stenosis, or any known cardiomyopathy etiology were excluded. The latter exclusion was inclusive of all patients receiving a final diagnosis of acute myocarditis, cardiac sarcoidosis, cardiac amyloidosis, hypertrophic cardiomyopathy, arrhythmogenic right ventricular cardiomyopathy (ARVC), restrictive cardiomyopathy (e.g.: cardiac amyloid), or constrictive pericardial disease. Only patients completing a minimum of 12-months clinical follow-up were considered eligible^18^.

The study was approved by the Conjoint Health Research Ethics Board at the University of Calgary and all subjects provided written informed consent. All research activities were performed in accordance with the Declaration of Helsinki.

### CMR imaging and analysis protocol

CMR imaging was performed using 3-Tesla clinical scanners (Prisma or Skyra, Siemens Healthcare, Erlangen, Germany), as previously described^18^. Standardized CMR imaging protocols were used, inclusive of cine imaging using a steady-state free precession (SSFP) pulse sequence in sequential short-axis views from above the pulmonary valve to beyond the cardiac apex. Long-axis imaging was performed in the two-, three- and four-chamber views. LGE imaging was performed using a standard inversion recovery gradient echo pulse sequence in matched views to cine imaging. LGE imaging was performed 10-min following intravenous administration of gadolinium contrast (0.1–0.2 mmol/kg; Gadovist; Bayer, Inc).

Quantitative image analysis was performed using commercially available software (cvi^42^; Circle Cardiovascular Imaging Inc., Calgary, Canada) with use of standardized operational procedures (SOP) adherent to published Society of Cardiovascular Magnetic Resonance (SCMR) recommendations^19^. Short-axis cine images were analyzed using semi-automated contour tracing of endocardial and epicardial borders to obtain the LV end-diastolic volume (LVEDV), LV end-systolic volume (LVESV), LV ejection fraction (LVEF), LV mass, RV end-diastolic volume (RVEDV), RV end-systolic volume (RVESV), and RV ejection fraction (RVEF). For the LV, papillary muscles were included in the LV mass and excluded from LV blood volumes. Left atrial (LA) volumes were measured at the LV end-systolic phase prior to mitral valve opening using the bi-plane area-length method from temporally matched 4- and 2-chamber cine views. All volumetric analyses were indexed to body surface area (BSA), where appropriate, using the Mosteller formula.

**Figure 1:**
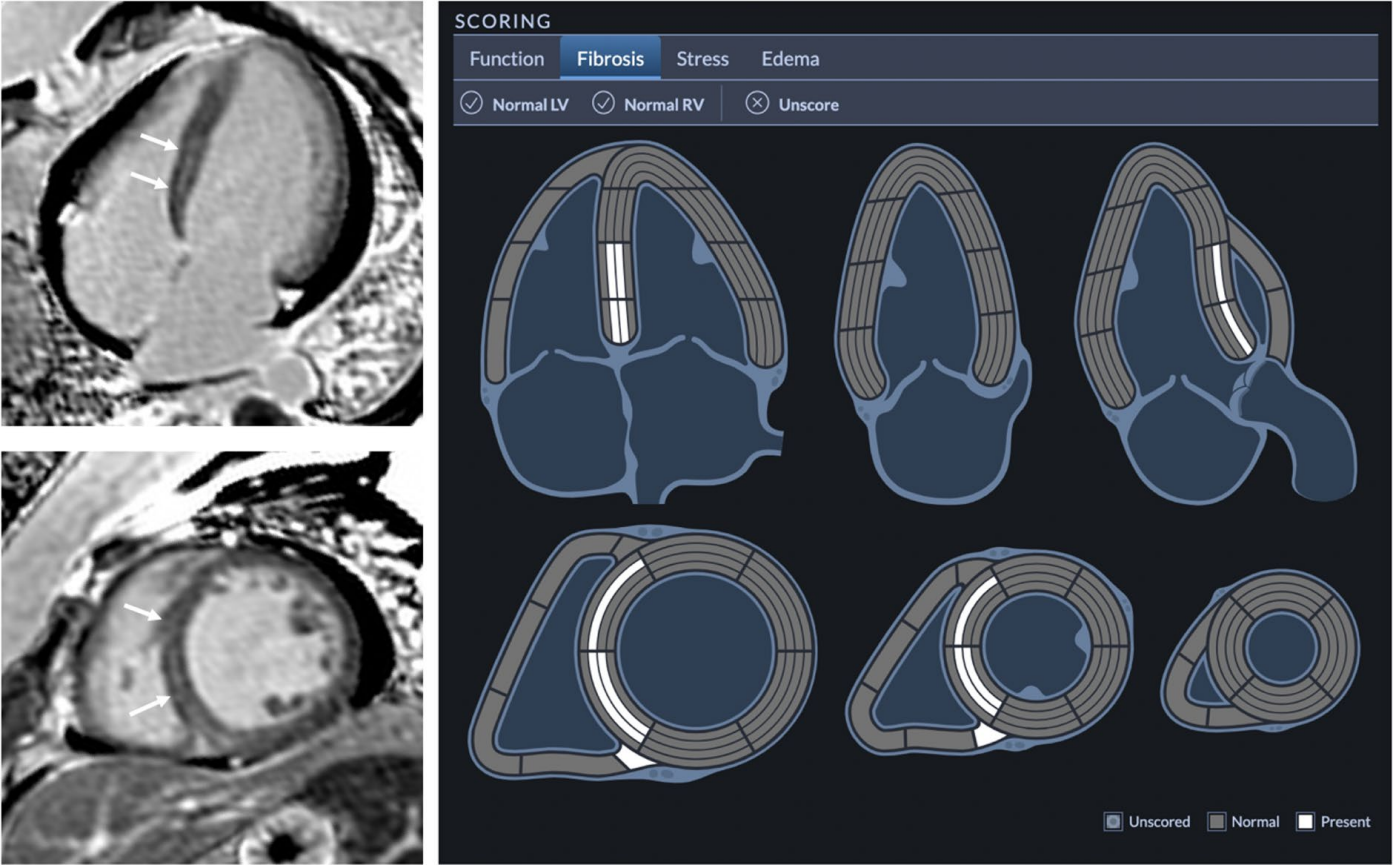
Example late gadolinium enhancement (LGE) reporting of mid-wall striae (MWS) pattern fibrosis in a 38-year-old female with dilated cardiomyopathy. Concurrent inferior right ventricular insert site fibrosis is also noted.

Standardized reporting (cardioDI™, Cohesic Inc, Calgary, Alberta) was used to collect all disease phenotype features, inclusive of regional patterns of replacement myocardial fibrosis. The presence of any myocardial fibrosis and patterns of fibrosis were coded, the latter described as: sub-endocardial, mid-wall striae (MWS), mid-wall patchy (MWP), sub-epicardial, and diffuse, as previously described^20^. All coding was performed by expert readers with a minimum of 5-years of clinical practice experience followed by blinded adjudication by a core laboratory reader (YM). For disagreement in pattern scoring the study was reviewed by a third expert reader (JW) to provide a final consensus coding. The spatial extent of fibrosis was coded for each of the 17 American Heart Association (AHA) segments using a sub-segmental model, each segment divided into 4 transmural zones, as previously validated^21^ and as shown in Fig. 1. MWS fibrosis was defined as the visual presence of a linear hyper-intensity of greatest intensity at the mid-myocardial transmural region of the interventricular septum seen in at least 2 contiguous or perpendicular imaging planes.

### Collection of clinical outcomes

The primary outcome was time to first incident HF admission, defined as occurrence of an ICD-10 coded heart failure admission (I50.X) followed by confirmation of hospitalization ≥ 24 h duration. Two composite secondary outcomes were selected: a HF-related outcome of HF admission, left ventricular assist device (LVAD) implantation, cardiac transplantation, or all-cause mortality; and an arrhythmia-related composite secondary outcome of appropriate ICD therapy, sudden cardiac death (SCD), survived sudden cardiac arrest (SCA), or sustained VT requiring cardioversion. Heart transplantation or LVAD occurrence was tracked by procedural ICD-10 coding. Device interrogations were blindly reviewed for confirmation of, and appropriateness of therapy delivered for ventricular fibrillation or fast VT (R-R interval < 320 ms). All-cause mortality was determined through the combined consideration of data from Vital Statistics Alberta and in-hospital coded death. All events were referenced to the date of index Cardiac MRI study.

### Statistical analysis

All descriptive statistics were expressed as mean ± standard deviation (SD) or median (interquartile range [IQR]). Categorical variables were expressed in counts with percentages. Missing laboratory or ECG data was accounted for by multiple imputation by chained reactions (MICE) with no more than 15% of missing values permitted for any variable entered into multivariate models. Comparison between 2 groups was performed with the independent Student *t* test or Mann–Whitney U test. Fisher exact or Chi-squared tests were used to compare categorical data. Univariable associations between demographic or CMR characteristics and clinical outcomes were performed using Cox proportional hazards regression. Variables that were statistically significant (*p* < 0.05) were considered eligible for inclusion into multivariate models. All eligible variables were tested for collinearity prior to being included in final multivariable models using stepwise Cox regression. This was restricted to eleven variables to avoid over-fitting and priority given to those with high univariable hazards or high perceived clinical value. Kaplan–Meier curves were generated for LVEF as a binary category with a cut off > 35%. Kaplan–Meier curves were also generated for strata consisting of combinations of LVEF and presence/absence of mid-wall striae (MWS). A competing risk analysis was performed using a sub-distribution hazards model (Fine-Gray model) to account for the effect of death on the primary outcome, as previously described^22,23^. Statistical analyses were performed using SPSS for Macintosh Version 26.0 (IBM Corp, Armonk NY) and R version 3.5.3.

### Ethical approval and consent to participate

The study was approved by the Conjoint Health Research Ethics Board at the University of Calgary and all subjects provided written informed consent. All research activities were performed in accordance with the Declaration of Helsinki.

### Consent for publication

All patients provided written informed consent.

## Results

### Baseline clinical characteristics

A total of 719 patients meeting study eligibility were prospectively recruited; their baseline characteristics described in Table 1. The median age was 57 (IQR 47–65) years old with a higher proportion of males. Thirty-five percent of patients had hypertension, 39% hyperlipidemia, and 15% were diabetic. Approximately one-third of patients had NYHA III/IV symptoms at time of enrolment.

**Table 1:** Baseline clinical, electrocardiographic and laboratory characteristics for the study population, and for those patients with and without the primary outcome of heart failure admission.

Variable	All subjects N = 719	Event – N = 615	Event + N = 104	*P* value	HR (95% CI) *p < 0.05
**Clinical characteristics**					
Age (years)	57 (19)	57 (18)	62 (17)	**0.011**	1.02 (1.00–1.03)*
Male sex *n,* (%)	516 (72)	448 (73)	68 (65)	0.126	0.72 (0.48–1.08)
BMI (m^2^)	28 (7)	28 (7)	29 (10)	0.90	1.03 (1.01–1.06)*
BSA (kg /m^2^)	2.1 (0.4)	2.1 (0.4)	2.1 (0.4)	0.669	1.23 (0.65–2.32)
Heart rate (bpm)	70 (20)	69 (20)	75 (19)	**0.001**	1.02 (1.01–1.03)*
Systolic BP (mmHg)	114 (22)	115 (23)	108 (24)	**0.001**	0.98 (0.97–0.99)*
Diastolic BP (mmHg)	69 (16)	69 (16)	66 (19)	0.079	0.99 (0.97–1.00)
Smoking (active) *n, *(%)	138 (19)	122 (20)	16 (15)	0.346	0.75 (0.44–1.28)
Diabetes mellitus *n, *(%)	109 (15)	78 (13)	31 (30)	**< 0.001**	2.75 (1.81–4.19)*
Hypertension *n, *(%)	251 (35)	203 (33)	48 (46)	**0.011**	1.62 (1.10–2.38)*
Hyperlipidemia *n, *(%)	283 (39)	230 (37)	53 (51)	**0.012**	1.70 (1.16–2.49)*
Atrial fibrillation n, (%)	140 (20)	114 (19)	26 (25)	0.140	1.44 (0.92–2.24)
NYHA Class III/IV *n*, (%)	201 (28)	157 (26)	44 (42)	**0.001**	2.09 (1.42–3.08)*
**Baseline medications**					
Beta blockers *n*, (%)	569 (79)	473 (77)	96 (92)	**< 0.001**	3.30 (1.60–6.79)*
ACEi or ARB *n*, (%)	563 (78)	468 (76)	95 (91)	**< 0.001**	3.10 (1.57–6.14)*
Entresto *n*, (%)	59 (8)	50 (8)	9 (9)	0.847	1.17 (0.59–2.32)
Loop diuretic *n*, (%)	197 (27)	131 (21)	66 (64)	**< 0.001**	5.31 (3.56–7.92)*
K^+^ sparing diuretic *n*, (%)	254 (35)	199 (32)	55 (53)	**< 0.001**	2.23 (1.52–3.28)*
Thiazide diuretic *n*, (%)	56 (8)	47 (8)	9 (9)	0.694	1.14 (0.57–2.25)
Lipid lowering (statin) *n*, (%)	267 (37)	216 (35)	51 (49)	**0.008**	1.72 (1.17–2.53)*
Digoxin *n*, (%)	60 (8)	40 (7)	20 (19)	**< 0.001**	2.87 (1.76–4.68)*
Anticoagulation *n*, (%)	184 (26)	136 (22)	48 (46)	**< 0.001**	2.90 (1.97–4.27)*
Anti-arrhythmic *n*, (%)	39 (5)	31 (5)	8 (8)	0.248	1.45 (0.71–2.98)
Ca^++^ channel blocker (non-dihydropyridines) *n*, (%)	25 (4)	19 (3)	6 (6)	0.240	1.78 (0.78–4.05)
**Electrocardiography (12 lead ECG)**					
PR interval (ms)	168 (32)	168 (33)	168 (34)	0.332	1.01 (1.00–1.02)
QRS duration (ms)	104 (48)	104 (48)	108 (48)	0.598	1.00 (1.00–1.01)
QTc (Bazzett) (ms)	462 ± 37	460 ± 37	467 ± 37	0.111	1.01 (1.00–1.01)
**Laboratory testing**					
Hemoglobin (g/L)	144 (23)	145 (20)	138 (30)	**0.014**	0.98 (0.97–1.00)*
Creatinine (µmol/L)	90 (29)	89 (28)	96 (39)	**0.016**	1.00 (1.00–1.00)
Sodium (mmol/L)	140 (4)	140 (3)	139 (5)	**0.022**	0.91 (0.86–0.97)*
NT-proBNP (ng/L)^a^	4002 (3642)	3515 (4449)	5348 (3148)	0.079	1.00 (1.00–1.00)
**CMR imaging characteristics**					
LVEF (%)	40 (18)	42 (16)	31 (19)	**< 0.001**	0.96 (0.94–0.97)*
LVEF ≤ 35% *n*, (%)	289 (40)	223 (36)	66 (64)	**< 0.001**	2.87 (1.92–4.27)*
LV EDV (ml)	218 (99)	212 (89)	256 (128)	**0.005**	1.00 (1.00–1.01)*
LV EDVi (ml/ m^2^)	104 (43)	103 (41)	117 (60)	**0.008**	1.01 (1.00–1.01)*
LV ESV (ml)	128 (84)	125 (73)	167 (128)	**< 0.001**	1.00 (1.00–1.01)*
LV ESVi (ml/m^2^)	62 (40)	60 (36)	77 (63)	**< 0.001**	1.01 (1.01–1.01)*
LV mass (g)	135 (63)	133 (59)	161 (67)	**< 0.001**	1.01 (1.00–1.01)*
Indexed LV mass (g/m^2^)	66 (27)	65 (25)	73 (35)	**< 0.001**	1.02 (1.01–1.02)*
RVEF (%)	47 (13)	48 (13)	43 (16)	**0.001**	0.97 (0.96–0.99)*
RV EDV (ml)	174 (77)	176 (75)	158 (92)	0.266	1.00 (1.00–1.00)
RV EDVi (ml/m^2^)	84 (34)	85 (32)	80 (38)	0.086	1.00 (0.99–1.01)
RV ESV (ml)	91 (59)	90 (57)	95 (73)	0.445	1.00 (1.00–1.01)
RV ESVi (ml/m^2^)	44 (26)	44 (24)	44 (29)	0.492	1.01(1.00–1.02)
LA vol (ml)	81 (47)	79 (43)	95 (57)	**< 0.001**	1.01 (1.01–1.01)*
Indexed LA vol (ml/m^2^)	40 (19)	39 (19)	45 (30)	**< 0.001**	1.02 (1.01–1.03)*
Any LGE (%)	228 (32)	182 (30)	46 (44)	0.004	1.75 (1.19–2.58)*
Mid-wall striae LGE *n*, (%)	178 (25)	136 (22)	42 (40)	< 0.001	2.14 (1.44–3.16)*
Mid-wall patchy LGE *n*, (%)	14 (2)	13 (2)	1 (1)	0.705	0.46 (0.06–3.30)
Sub-epicardial LGE *n*, (%)	60 (8)	52 (9)	8 (8)	1	0.93 (0.45–1.92)
Diffuse LGE *n*, (%)	3 (0.4)	2 (0.3)	1 n	0.375	2.15 (0.30 – 15.41)

Results of univariable regression analysis is shown for associations with the primary outcome.

*BMI* body mass index, *BSA* body surface area, *NYHA* New York heart association, *ACE-I* angiotensin converting enzyme inhibitor, *ARB* angiotensin II receptor antagonist, *LVEF* left ventricular ejection fraction, *LVEDV* left ventricular end diastolic volume, *LVEDVi* indexed left ventricular diastolic volume, *LVESV* left ventricular end systolic volume, *LVESVi* indexed left ventricular end systolic volume, *RVEF* right ventricular ejection fraction, *RVEDV* right ventricular end diastolic volume, *RVEDVi* indexed right ventricular end diastolic volume, *RVESV* right ventricular end systolic volume, *RVESVi* indexed right ventricular end systolic volume, *LA vol* left atrial volume, *LGE* late gadolinium enhancement.

^a^NTproBNP was clinically performed in 208 subjects at time of CMR imaging.

The "bold, asterisk" means that those hazard ratios are statistically significant with a *p* value of < 0.05.

### Baseline magnetic resonance imaging characteristics

The median LVEF was 40% (IQR 29–47%, range 9–50%) with 289 patients (40%) having an LVEF ≤ 35%. The median non-indexed LV end diastolic volume (LVEDV) was 218 (IQR 176–275) ml with a body surface area (BSA) indexed value of 104 (IQR 87–130) ml/m^2^. The non-indexed and indexed LV mass were 135 g (IQR 108–171 g) and 66 g/m^2^ (IQR 53–80 g/m^2^), respectively. The median RVEF was 47% (IQR 40–53%, range 12–75%) with a median RV end diastolic volume (RVEDV) of 174 ml (IQR 139–216 ml) and BSA-indexed value of 84 ml/m^2^ (IQR 69–103) ml/m^2^. The median non-indexed LA volume was 81 ml (IQR 61–108 ml) with an indexed LA volume of 40 ml/m^2^ (IQR 31–50 ml/m^2^).

LGE imaging demonstrated 228 patients (32%) had any LGE abnormality. A total of 178 patients (25%) demonstrated a MWS pattern, 60 (8%) a sub-epicardial pattern, 14 (2%) a mid-wall patchy pattern, and 3 (0.4%) a diffuse pattern. Example images and coding are provided in Fig. 1.

### Population differences according to LVEF-based stratification

Stratification by conventional LVEF-based risk thresholds for “severe” LV dysfunction resulted in 289 patients (40%) with LVEF ≤ 35% and 430 patients (60%) with LVEF 35–49%. Significant differences between these cohorts are shown in Supplemental Table 1. Patients with LVEF ≤ 35% were older, more likely diabetic, and described worse symptom burden by NYHA status. Corresponding MRI measurements showed higher biventricular volumes with a higher prevalence of MWS fibrosis (37% vs 16%, *p* < 0.001). ACE-inhibitors/ARB, beta-blocker and loop diuretic use was also higher among patients with LVEF ≤ 35%.

### Population differences according to fibrosis phenotype stratification

Stratification by fibrosis phenotype resulted in 178 patients (25%) with MWS and 541 patients (75%) without. Significant differences between these cohorts are shown in Supplemental Table 2. Patients with MWS were older and described a worse symptom burden by NYHA status. Corresponding MRI measurements showed higher left ventricular volumes and higher prevalence of LVEF ≤ 35% among patients with MWS (61% vs 34%, *p* < 0.001). Patients with MWS had significantly greater use of ACE-inhibitors/ARB and loop diuretics compared to patients without.

### Associations with the primary outcome

Over a median follow up of 1044 (IQR 721–136) days, 104 patients (15%) experienced the primary outcome of incident HF admission. Univariable analysis identified a number of baseline clinical variables associated with this primary outcome, inclusive of age, BMI, heart rate, systolic BP, diabetes, hypertension, hyperlipidemia, NYHA class, hemoglobin, and serum sodium (Table 1). Similarly, numerous MRI-based characteristics were associated with the primary outcome, including LVEF, LVEDVi, LVESVi, LV indexed mass, RVEF, indexed LA volume and MWS fibrosis. As shown in Table 1, the unadjusted hazard associated with LVEF ≤ 35% was 2.87 (95% CI 1.92–4.27). The respective hazard observed for MWS was 2.14 (95% CI 1.44–3.16).

Stepwise multivariable models were constructed to identify the independent predictive value of MWS fibrosis for the primary outcome. Following adjustment for age, sex, diabetes, hypertension, NYHA III/IV, ACE-inhibitor/ARB use, beta-blocker use, LVEF, RVEF and indexed LV mass, the presence of MWS fibrosis remained an independent predictor of incident HF admission, providing a hazard ratio of 1.65 (95% CI 1.11–2.47) (Table 2). In this model, diabetes remained a strong predictor, providing a 2.45-fold risk of HF admission (95% CI 1.59–3.77). Other independent predictors were female gender, NYHA III/IV symptom status, beta-blocker use, worsening LVEF and indexed LV mass. A competing risk analysis was performed to adjust for the interval occurrence of death and its influence on the primary outcome. In this model, MWS fibrosis remained an independent predictor of the primary outcome with a HR of 1.57 (95% CI 1.04–2.37).

**Table 2:** Multivariable analysis performed for the prediction of the primary outcome.

Variable	Hazard ratio (95% CI)	*P*
Sex (male)	0.59 (0.39–0.90)	0.014
Diabetes	2.45 (1.59–3.77)	< 0.001
NYHA III/IV	1.62 (1.08–2.43)	0.019
Beta Blocker	2.08 (1.00– 4.33)	0.05
LVEF (per 1% increase)	0.98 (0.96–1.00)	0.012
Indexed LV mass (per 1 g/m^2^ increase)	1.01 (1.00–1.02)	0.004
MWS pattern fibrosis	1.65 (1.11–2.47)	0.014

Variables included in the stepwise multivariate model include age, sex, body mass index, diabetes, hypertension, NYHA III/IV, ACE-inhibitor/ARB use, beta-blocker use, LVEF, RVEF, indexed LV mass and MWS fibrosis.

*NYHA* New York heart association, *LVEF* left ventricular ejection fraction, *MWS* mid-wall striae.

**Figure 2:**
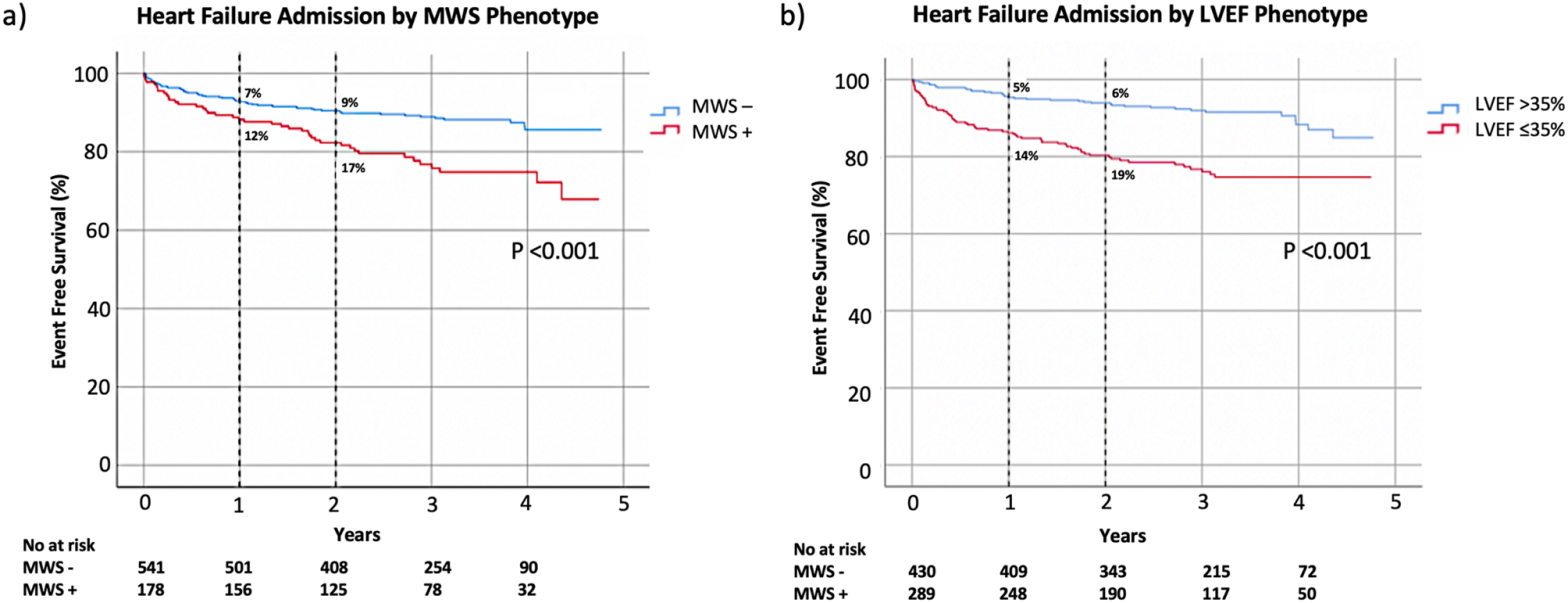
Kaplan–Meier event free survival curve for the primary outcome of heart failure hospitalization in patients with and without (**a**) mid-wall striae (MWS) fibrosis, and (**b**) LVEF ≤ 35%.

**Figure 3:**
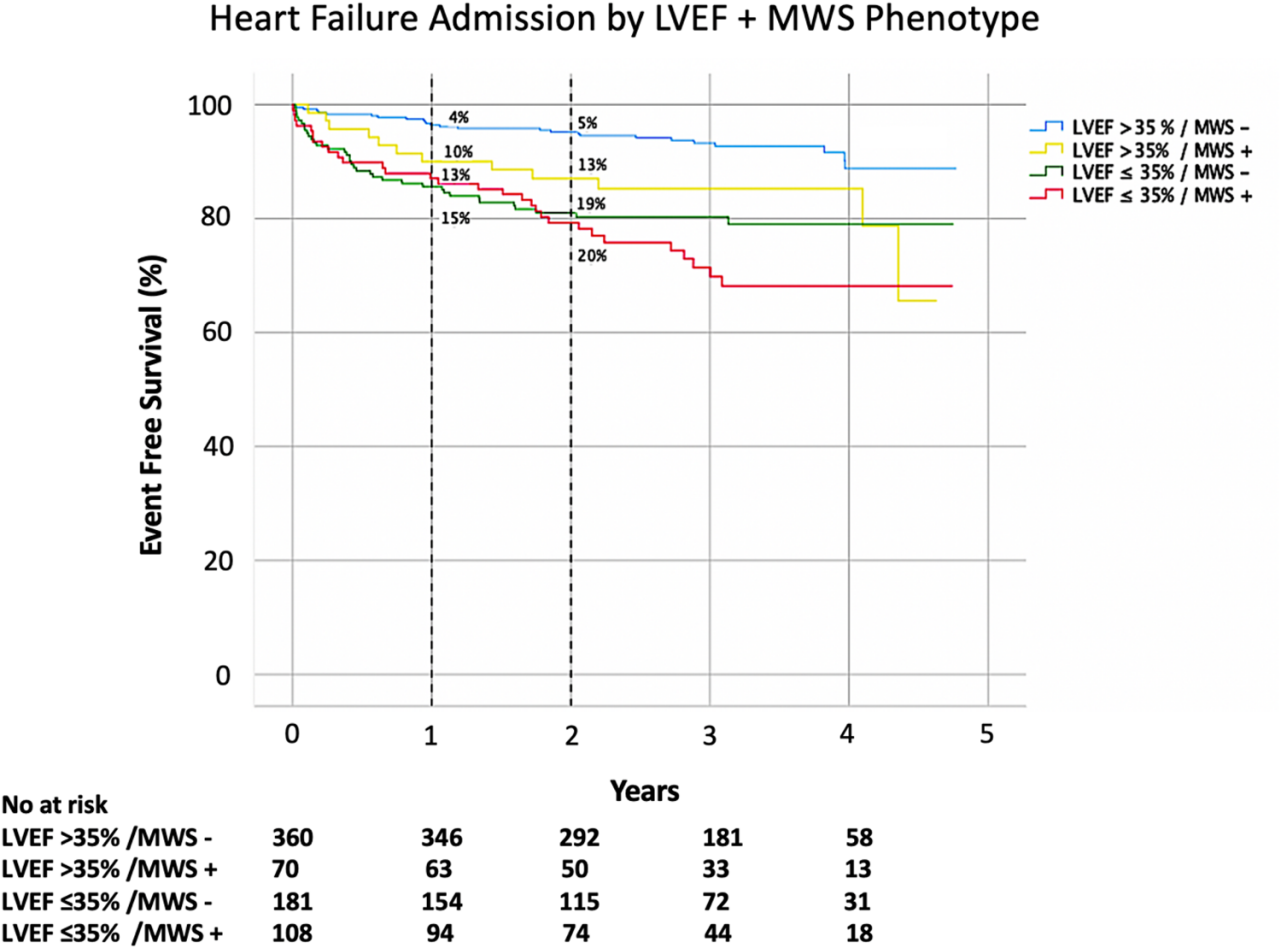
Kaplan–Meier event free survival curve for the primary outcome of heart failure hospitalization stratified by the combined presence or absence of mid-wall striae (MWS) fibrosis and LVEF ≤ 35%.

**Figure 4:**
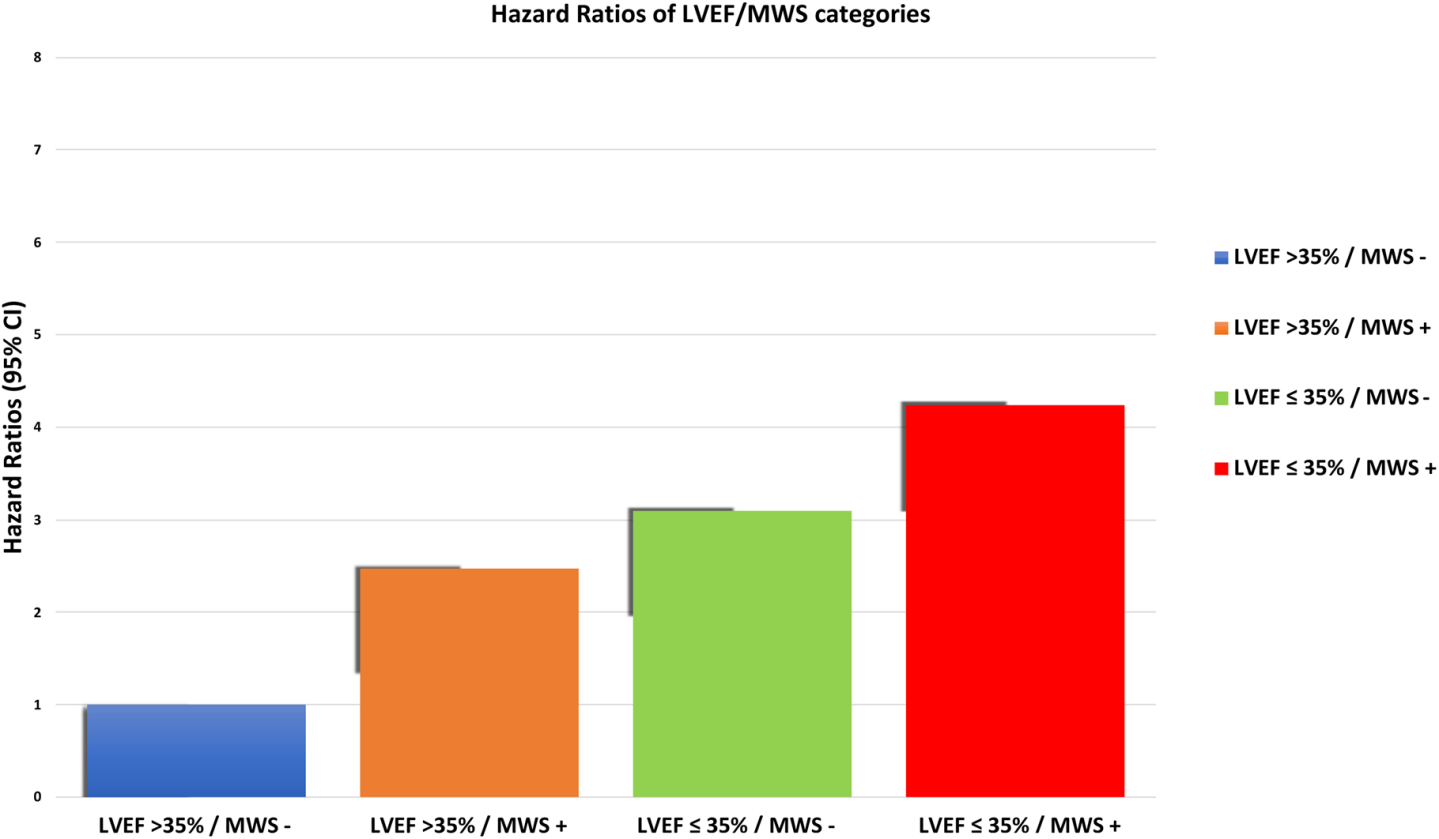
Hazard ratios provided by each of four DCM phenotypes defined using the combined presence or absence of mid-wall striae (MWS) fibrosis and LVEF ≤ 35%.

Kaplan–Meier analysis was performed to evaluate event-free survival in patients with and without MWS (Fig. 2a). The cumulative risk of HF admission at 1-year was 12% in those with MWS versus 7% in those without (*p* < 0.001). Similar analysis stratified by LVEF ≤ 35% showed 1-year cumulative event rates of 14% and 5%, respectively (*p* < 0.001), as shown in Fig. 2b. Given independent utility demonstrated in multivariable analysis we then assessed their combined value, as shown in Fig. 3. This demonstrated patients with LVEF > 35% plus MWS experienced risk equivalent to those with an LVEF ≤ 35%. Respective hazards for each of the combined phenotype categories is provided in Fig. 4.

Finally, separate multivariable models were constructed to assess the prognostic value of MWS in patients with and without LVEF ≤ 35% (66 and 38 events, respectively). Among those with LVEF > 35%, MWS provided an adjusted hazard of 2.40 (95% CI 1.20–4.78), with male sex, hypertension and RVEF remaining significant predictors. Among patients with LVEF ≤ 35%, MWS did not remain significant, while diabetes remained a strong predictor (HR 2.44, 95% CI 1.45–4.11) followed by NYHA class (HR 1.69, 95% CI 1.04–2.74) (Table 3).

**Table 3:** Multivariable analysis performed for the prediction of the primary outcome in (1) sub-group of patients with LVEF ≤ 35%, (2) sub-group of patients with LVEF > 35%.

LVEF strata	Hazard ratio (95% CI)	*P*
**LVEF ≤ 35%**		
Diabetes	2.44 (1.45–4.11)	**0.001**
NYHA III/IV	1.69 (1.04–2.74)	**0.035**
LV indexed mass (per 1 g/m^2^ increase)	1.01 (1.00–1.02)	0.081
**LVEF > 35%**		
Sex (male)	0.28 (0.14–0.55)	**< 0.001**
Diabetes	2.06 (0.974–4.37)	0.06
Hypertension	1.99 (1.01–3.91)	**0.05**
ACE-i/ARB	2.38 (0.81–6.96)	0.115
RVEF (per 1% increase)	0.95 (0.91–0.98)	**0.006**
MWS pattern fibrosis	2.40 (1.20–4.78)	**0.013**

Variables included in the stepwise multivariate model include age, sex, body mass index, diabetes, hypertension, NYHA III/IV, ACE-inhibitor/ARB use, beta-blocker use, LVEF, RVEF, indexed LV mass and MWS fibrosis.

*NYHA* New York heart association, *RVEF* right ventricular ejection fraction, *MWS* mid-wall fibrosis.

The "bold" means that the *p*-value is statistically significant at < 0.05.

### Associations with the secondary composite heart failure outcome

During the follow-up period a total of 127 patients (18%) experienced the secondary composite outcome. The first registered event was HF admission in 103 patients, all-cause death in 23 patients, and LVAD implantation in 1 patient. No patients underwent cardiac transplantation.

Univariable analysis demonstrated numerous clinical and MRI-based variables associated with the composite outcome, as shown in Supplementary Table 3. MWS was associated with an unadjusted hazard of 2.15 (95% CI 1.50–3.06) while LVEF ≤ 35% provided a hazard of 2.40 (95% CI 1.69–3.43).

Stepwise multivariable analysis demonstrated MWS to be independently associated with the composite secondary outcome following adjustment for age, sex, diabetes, hypertension, NYHA III/IV, ACE-inhibitor/ARB use, beta-blocker use, LVEF, RVEF and indexed LV mass, providing a 1.80-fold increased risk (95% CI 1.26–2.58). LVEF was not an independent predictor of the secondary outcome in multivariate analysis.

**Figure 5:**
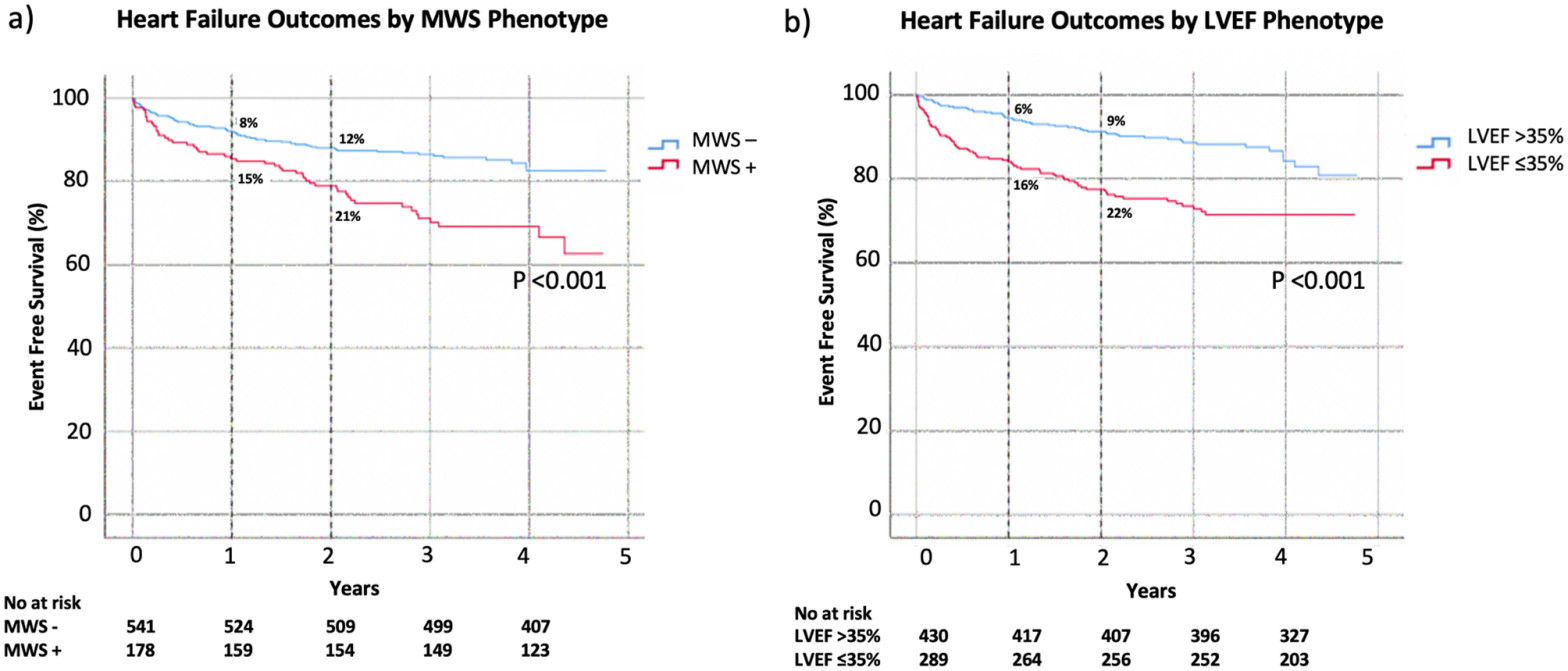
Kaplan–Meier event free survival curve for the secondary heart failure composite outcome of heart failure hospitalization in patients with and without (**a**) mid-wall striae (MWS) fibrosis, and (**b**) LVEF ≤ 35%.

**Figure 6:**
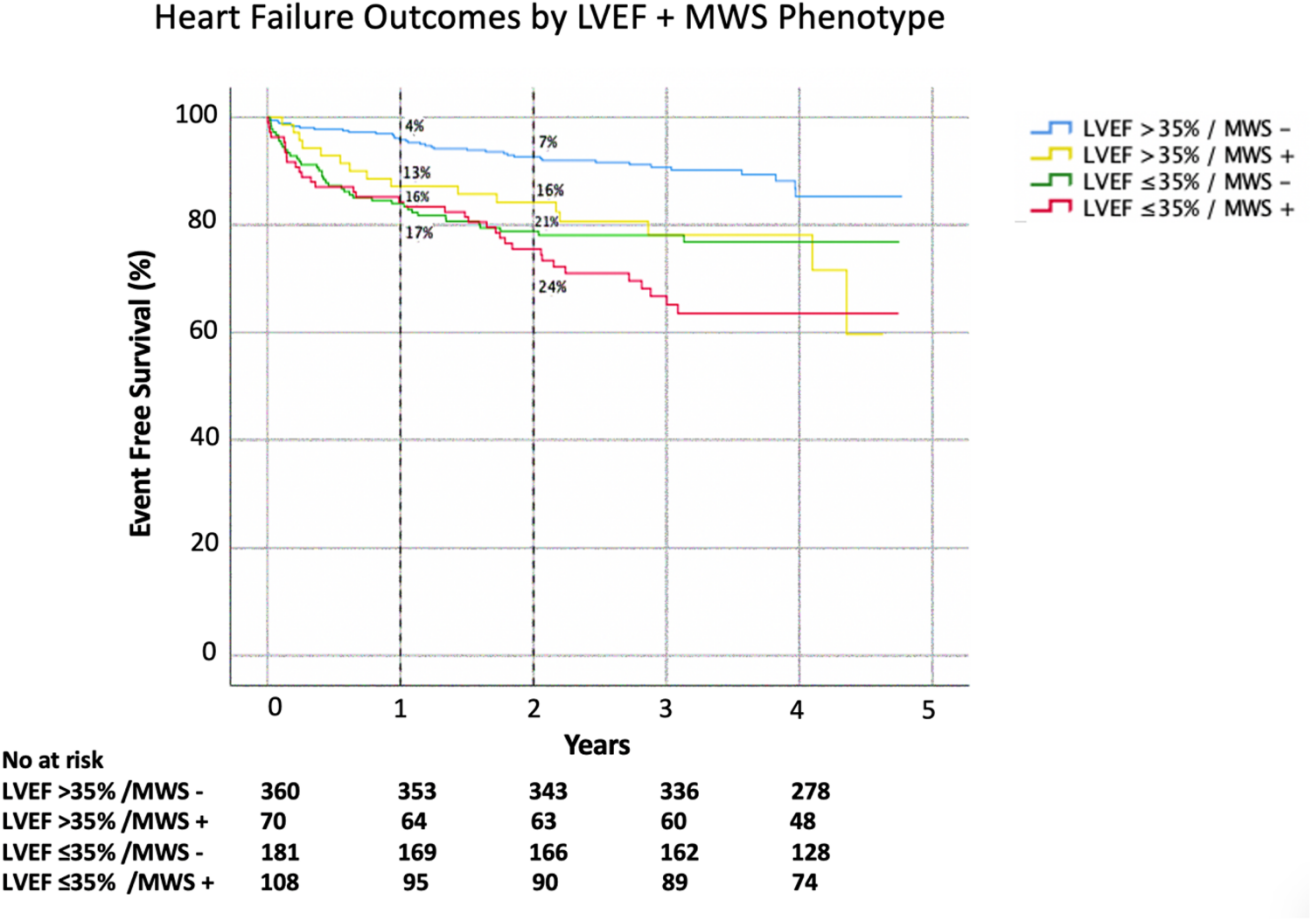
Kaplan–Meier event free survival curve for the secondary heart failure outcome of HF admission, left ventricular assist device (LVAD) implantation, cardiac transplantation or all-cause mortality stratified by the combined presence or absence of mid-wall striae (MWS) fibrosis and LVEF ≤ 35%.

Kaplan–Meier analysis for occurrence of the secondary outcome in patients with versus without MWS fibrosis is shown in Fig. 5a. Respective cumulative risks of the secondary outcome at 1 year were 15% and 8% (*p* < 0.001). One-year cumulative event rates in patients with versus without LVEF ≤ 35% were 16% and 6%, respectively (*p* < 0.001), as shown in Fig. 5b. The combined phenotype of LVEF > 35% plus MWS was associated with a 1-year cumulative event rate equivalent to those with LVEF ≤ 35% (Fig. 6).

### Associations with the secondary composite arrhythmia outcome

A total of 112 cardiac devices were implanted, consisting of 95 ICD or CRT-D devices, 3 CRT-P devices, and 14 permanent pacemakers. A total of 45 patients experienced one or more ventricular arrhythmia clinical outcomes. The first documented event was appropriate ICD therapy in 21, SCD in 6, survived SCA in 13, and sustained VT requiring DC cardioversion in 11. Six individuals experienced SCD, all having a prior coded arrhythmic event.

Univariable analysis demonstrated numerous clinical and MRI-based variables associated with this composite outcome, as shown in Supplementary Table 4. MWS was associated with an unadjusted hazard of 2.31 (95% CI 1.28–4.17) while LVEF ≤ 35% provided an unadjusted hazard of 1.93 (95% CI 1.07–3.48).

Stepwise multivariable analysis demonstrated MWS to be independently associated with the composite secondary arrhythmic outcome following adjustment for age, sex, NYHA III/IV and LVEF, providing a 2.23-fold increased risk (95% CI 1.23–4.03). LVEF was not an independent predictor of the secondary outcome in multivariate analysis.

**Figure 7:**
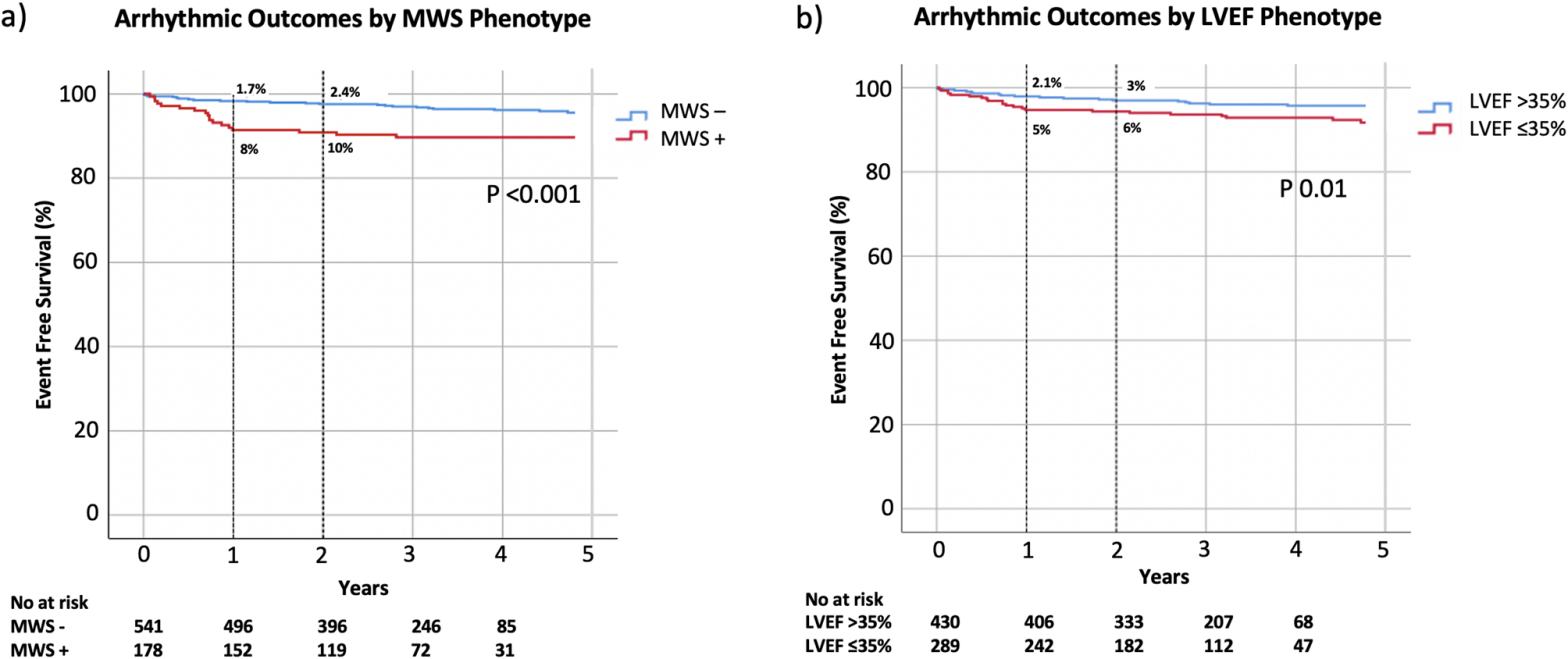
Kaplan–Meier event free survival curve for the secondary arrhythmic composite outcome of appropriate ICD therapy, sudden cardiac death (SCD), survived sudden cardiac arrest (SCA), or sustained VT requiring cardioversion with and without (**a**) mid-wall striae (MWS) fibrosis, and (**b**) LVEF ≤ 35%.

**Figure 8:**
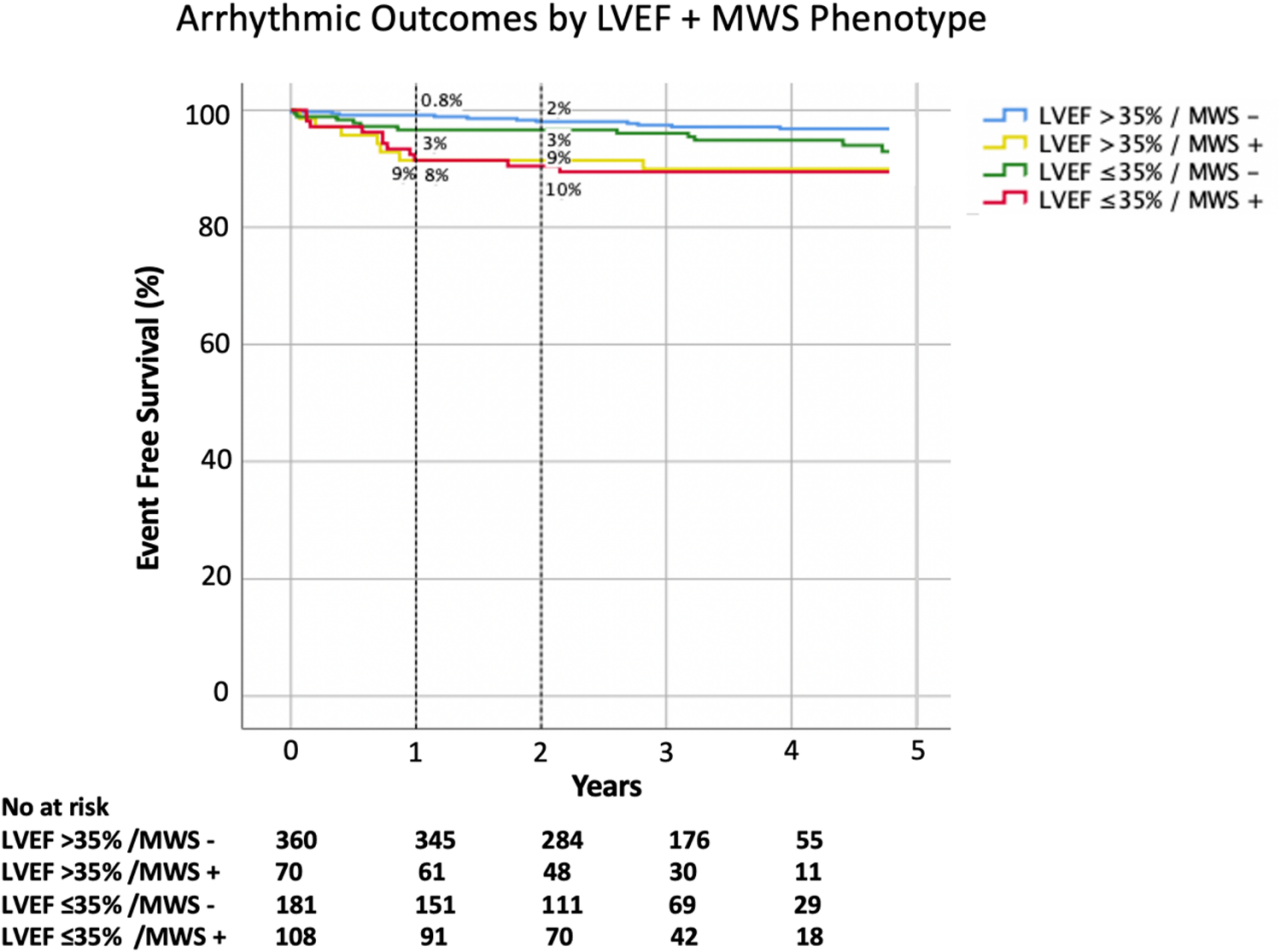
Kaplan–Meier event free survival curve for the secondary arrhythmic outcome of appropriate ICD therapy, sudden cardiac death (SCD), survived sudden cardiac arrest (SCA), or sustained VT requiring cardioversion stratified by the combined presence or absence of mid-wall striae (MWS) fibrosis and LVEF ≤ 35%.

Kaplan–Meier analysis for occurrence of the secondary arrhythmic outcome in patients with versus without MWS fibrosis is shown in Fig. 7. Respective cumulative risks of the secondary arrhythmic outcome at 1 year were 1.7% and 8% (*p* < 0.001). One-year cumulative event rates in patients with versus without LVEF ≤ 35% were 5% and 2%, respectively (*p* = 0.025). The combined phenotype of LVEF > 35% plus MWS was associated with a 1-year cumulative event rate equivalent to those with LVEF ≤ 35% (*p* = 0.732) (Fig. 8).

## Discussion

This study was dedicated to assessing the influence of mid-wall striae fibrosis on incident HF admission rates in patients referred for the CMR evaluation of DCM. Our findings demonstrate MWS pattern fibrosis to be a strong predictor of future HF admission independent of confounding variables, inclusive of LVEF. Among those with an LVEF above 35%, MWS was observed in 16% of patients and was associated with event risks equivalent to those with LVEF ≤ 35%.

Global estimates of LV function (i.e. LVEF) remain the dominate phenotypic arbitrator of therapeutic decisions in the management of systolic heart failure^5,24–27^. However, solitary use of this crude phenotypic marker is increasingly being challenged by the expanding availability of advanced phenotypic markers predictive of major cardiovascular outcomes in this population. Although commonly managed as a singular disease entity, DCM is increasingly recognized to have complex pathophysiology with a broad range of phenotypic expression (Prasad review paper). Cardiac MRI has become increasingly engaged for characterizing unique DCM phenotypes, both for the purposes of excluding known causative states (e.g. cardiac sarcoidosis) and for identifying unique patterns of adverse remodelling. The latter has led to the identification of DCM sub-cohorts demonstrating advanced degrees of replacement fibrosis on LGE imaging in a septal striae patter, this finding consistently associated with greater risk of sudden cardiac arrest (SCA) or appropriate implantable cardioverter-defibrillator (ICD) therapy^6–10,14–16^. However, a number of studies have similarly shown prognostic value for non-arrhythmic clinical outcomes, including cardiovascular mortality or transplantation, and broader composite endpoints inclusive, but not focussed on, HF hospitalization^6,8,10,12,28,29^. In a study of 472 DCM patients Gulati, et al*.* demonstrated that the presence of MWS pattern fibrosis was associated a 2.43-fold (95% CI 1.50–3.92) increased risk of all-cause mortality, and a borderline increased risk (HR 1.62, 95% CI 1.0–2.61) of the composite endpoint of HF death, hospitalization or transplantation^6^. The same group subsequently studied a composite endpoint inclusive of all-cause mortality, heart failure hospitalization and aborted SCA among 120 patients with new-onset DCM, describing a 2.97-fold (95% CI 1.37–6.45) increased risk in those with MWS^29^. Combined consideration of all studies including HF admission within composite clinical endpoints has allowed for meta-analyses to explore the potential value of abnormal LGE (multiple employed criteria) to identify patients at elevated risk of HF admission^30–34^. For example, a meta-analysis by Becker et al*.* estimated an unadjusted 2.66-fold risk of future HF admission (95% CI 1.67–4.24) among patients with MWS^25^. The current study provided a sufficient population size with cumulative clinical events to appropriately examine this association in the context of multivariable adjustment, demonstrating that MWS presence is associated with independent risk of HF admission in this referral cohort.

A critical observation from this study was the capacity of LGE-MRI to identify DCM patients with mild-moderate LV dysfunction (LVEF 36–50%) who experience equivalent HF event rates to those with severe LV dysfunction (LVEF ≤ 35%). Approximately 1 of 6 patients (16%) with mild-moderate LV dysfunction showed MWS fibrosis and experienced incident event rates similar to those with LVEF ≤ 35%. This was similarly observed for the secondary arrhythmic outcome: those patients with LVEF > 35% and MWS have an identical event curve to those with LVEF < 35% without MWS. This demonstrates the potential for fibrosis-based phenotyping to significantly expand DCM population eligibility for intensive HF therapeutic strategies and primary prevention ICD.

Our study’s unique focus on the primary outcome of HF admission recognizes the strong influence of this event has on patient morbidity and healthcare resource consumption. Each HF admission is estimated to incur a median cost of $14,621 USD and associated with repeat admission at 60 days in one-third of patients^35^. Further, occurrence of HF admission has been associated with elevated future risk of mortality, this highlighted by Blackledge et al.^36^ who described a 1-year mortality rate of 43% among patients admitted for decompensated HF; climbing to 73% at 5-years. Similar findings were confirmed in two other large cohort studies^37,38^. Accordingly, validation of diagnostic markers with capacity to identify patients at high risk of this clinical outcome delivers expanded opportunity for personalized cardiovascular care strategies.

### Limitations

This study was prospectively conducted at two associated hospitals within a single tertiary care healthcare system. Accordingly, our study population may suffer from regional practice bias and would benefit from external validation. Invasive coronary angiography was not mandated in this study and was conducted in accordance with justifiable clinical need. Patients not undergoing invasive angiography were considered to have a non-ischemic etiology on the basis of composite clinical history (i.e. lack of prior myocardial infarction or revascularization) in combination with absence of subendocardial pattern injury on LGE imaging. This approach introduces the potential for an exclusion of DCM patients with incidental ischemic injury patterns (i.e. embolic infarction), however is considered a conservative approach for the exclusion of CAD-related cardiomyopathy justified by prior cohort studies^7,15^. Signal threshold-based quantification of replacement fibrosis volume was not performed. Therefore, a comparison of fibrosis extent by this technique versus the binary classification of fibrosis patterns was not feasible. Finally, as an imaging service-based Registry, serum BNP levels were not consistently ordered and collected within close temporal association to CMR imaging: being captured within 3-months in only 208 subjects. Accordingly, we were unable to include this serum-based heart risk marker in multi-variable analyses.

## Conclusions

In a large cohort of patients referred to CMR for evaluation of DCM we demonstrated MWS fibrosis to be a powerful and independent predictor of HF admission, identifying patients at approximately a two-fold elevated risk. This risk was independent of LVEF and permitted the identification of patients with intermediate range LV dysfunction (LVEF 36–50%) who experience equivalent event rates to those with severe LV dysfunction (LVEF ≤ 35%). Similar value was observed for composite endpoints related to both HF and arrhythmia-focussed outcomes. Future randomized controlled trials aimed at expanded the use of intensive heart failure therapies for DCM populations with intermediate range LVEF and MWS fibrosis are warranted.

## Supplementary Information

Supplementary Tables.

## Data Availability

The datasets used and/or analysed during the current study are available from the corresponding author on reasonable request.

## Abbreviations

BSA: Body surface area
CABG: Coronary artery bypass grafting
CIROC: Cardiovascular Imaging Registry of Calgary
CMR: Cardiovascular magnetic resonance imaging
DCM: Dilated cardiomyopathy
EACVI: European Association of Cardiovascular Imaging
FDA: Food and Drug Administration
GFR: Glomerular Filtration Rate
HFrEF: Heart Failure with Reduced Ejection Fraction
HFpEF: Heart Failure with Preserved Ejection Fraction
ICD-10: International Classification of Diseases-10
ICD: Implantable Cardioverter Defibrillator
LGE: Late gadolinium enhancement
MACE: Major adverse cardiovascular events
NCT: ClinicalTrials.gov Identifier
NYHA: New York Heart Association
NICM: Non-ischemic cardiomyopathy
