# Supplementary material for: Mid-wall striae fibrosis predicts heart failure admission, composite heart failure events, and life-threatening arrhythmias in dilated cardiomyopathy
Source: Sci Rep. 2022 Feb 2;12:1739. doi: 10.1038/s41598-022-05790-y (PMC8810767; doi:10.1038/s41598-022-05790-y)
Supplement: Supplementary file 1 — Supplementary Tables. [file 41598_2022_5790_MOESM1_ESM.docx]

**Supplemental Table 1.** Baseline clinical, electrocardiographic and laboratory characteristics for the study population, and for those patients with and without an LVEF ≤35%.

| Variable | All subjects  N = 719 | LVEF ≤35%  N= 289 | LVEF >35%  N = 430 | P value |
| --- | --- | --- | --- | --- |
| Clinical characteristics | | | | |
| Age (years) | 57 (19) | 59 (18) | 56 (19) | **<0.001** |
| Male sex *n* (%) | 516 (72) | 213 (74) | 303 (71) | 0.354 |
| BMI (kg/m^2^) | 28 (7) | 28 (8) | 28 (7) | 0.417 |
| BSA (kg /m^2^) | 2.1 (0.4) | 2.1 (0.5) | 2.1 (0.3) | 0.892 |
| Heart rate (bpm) | 70 (20) | 73 (21) | 68 (19) | **<0.001** |
| Systolic BP (mmHg) | 114 (22) | 108 (25) | 116 (21) | **<0.001** |
| Diastolic BP (mmHg) | 69 (16) | 67 (17) | 71 (16) | **0.002** |
| Smoking (active) *n, (%)* | 138 (19) | 51 (18) | 87 (20) | 0.440 |
| Diabetes Mellitus *n, (%)* | 109 (15) | 54 (19) | 55 (13) | **0.034** |
| Hypertension *n, (%)* | 251 (35) | 104 (36) | 147 (34) | 0.633 |
| Hyperlipidemia *n, (%)* | 283 (39) | 116 (40) | 167 (39) | 0.756 |
| Atrial Fibrillation n, (%) | 140 (20) | 54 (19) | 86 (20) | 0.701 |
| NYHA Class III / IV *n, (%)* | 201 (28) | 112 (39) | 89 (21) | **<0.001** |
| Baseline Medications | | | | |
| Beta blockers *n, (%)* | 569 (79) | 258 (89) | 311 (72) | **<0.001** |
| ACEi or ARB *n, (%)* | 563 (78) | 258 (89) | 305 (71) | **<0.001** |
| Entresto *n, (%)* | 59 (8) | 42 (15) | 17 (4) | **<0.001** |
| Loop diuretic *n, (%)* | 197 (27) | 128 (44) | 69 (16) | **<0.001** |
| K^+^ sparing diuretic *n, (%)* | 254 (35) | 173 (60) | 81 (19) | **<0.001** |
| Thiazide diuretic *n, (%)* | 56 (8) | 23 (8) | 33 (8) | 0.89 |
| Lipid lowering (statin) *n, (%)* | 267 (37) | 109 (38) | 158 (37) | 0.814 |
| Digoxin *n, (%)* | 60 (8) | 38 (13) | 22(5) | **<0.001** |
| Anticoagulation *n, (%)* | 184 (26) | 88 (31) | 96 (22) | **0.018** |
| Anti-arrhythmic *n, (%)* | 39 (5) | 20 (7) | 19 (4) | 0.179 |
| Ca^++^ channel blocker (non-dihydropyridines) *n, (%)* | 25 (4) | 6 (2) | 19 (4) | 0.101 |
|  | | | | |
| PR interval (ms) | 168 (32) | 170 (32) | 164 (36) | 0.359 |
| QRS duration (ms) | 104 (48) | 108 (52) | 100 (42) | **0.001** |
| QTc (Bazzett) (ms) | 462 ± 37 | 475 ± 36 | 451 ± 36 | **<0.001** |
| Laboratory testing | | | | |
| Haemoglobin (g/L) | 144 (23) | 144 (24) | 143 (21) | 0.471 |
| Creatinine (µmol/L) | 90 (29) | 95 (33) | 87 (25) | **<0.001** |
| Sodium (mmol/L) | 140 (4) | 139 (4) | 140 (4) | **0.005** |
| CMR Imaging Characteristics | | | | |
| LVEF (%) | 40 (18) | 27 (12) | 45 (6) | **<0.001** |
| LV EDV (ml) | 218 (99) | 271 (123) | 191 (69) | **<0.001** |
| LV EDVi (ml/m^2^) | 104 (43) | 134 (55) | 95 (27) | **<0.001** |
| LV ESV (ml) | 128 (84) | 199 (110) | 106 (40) | **<0.001** |
| LV ESVi (ml/m^2^) | 62 (40) | 98 (48) | 52 (17) | **<0.001** |
| LV mass (g) | 135 (63) | 158 (65) | 123 (52) | **<0.001** |
| Indexed LV mass (g/m^2^) | 66 (27) | 76 (30) | 60 (22) | **<0.001** |
| RVEF (%) | 47 (13) | 41 (23) | 49 (11) | **<0.001** |
| RV EDV (ml) | 174 (77) | 174 (83) | 174 (72) | 0.422 |
| RV EDVi (ml/m^2^) | 84 (34) | 84 (36) | 84 (31) | 0.464 |
| RV ESV (ml) | 91 (59) | 101 (77) | 86 (49) | **<0.001** |
| RV ESVi (ml/m^2^) | 44 (26) | 48 (35) | 42 (21) | **<0.001** |
| LA vol (ml) | 81 (47) | 92 (56) | 75 (40) | **<0.001** |
| Indexed LA vol (ml/m^2^) | 40 (19) | 46 (24) | 37 (17) | **<0.001** |
| Any LGE (%) | 228 (32) | 123 (43) | 105 (24) | **<0.001** |
| Mid-wall striae LGE *n, (%)* | 178 (25) | 108 (37) | 70 (16) | **<0.001** |
| Mid-wall patchy LGE *n, (%)* | 14 (2) | 5 (2) | 9 (2) | 0.791 |
| Sub-epicardial LGE *n, (%)* | 60 (8) | 18 (6) | 42 (10) | 0.1 |
| Diffuse LGE *n, (%)* | 3 (0.4) | 3 (1) | 0 | 0.065 |

*BMI (Body Mass Index), BSA (Body Surface Area), NYHA (New York Heart Association), ACE-I (angiotensin converting enzyme inhibitor), ARB (angiotensin II receptor antagonist), LVEF (left ventricular ejection fraction), LVEDV (left ventricular end diastolic volume), LVEDVi (indexed left ventricular diastolic volume), LVESV (left ventricular end systolic volume), LVESVi (indexed left ventricular end systolic volume), RVEF (right ventricular ejection fraction), RVEDV (right ventricular end diastolic volume), RVEDVi (indexed right ventricular end diastolic volume), RVESV (right ventricular end systolic volume), RVESVi (indexed right ventricular end systolic volume, LA vol (left atrial volume), LGE (late gadolinium enhancement).*

**Supplemental Table 2.** Baseline clinical, electrocardiographic and laboratory characteristics for the study population, and for those patients with and without mid-wall striae (MWS) pattern fibrosis.

| Variable | All subjects  N = 719 | MWS -  N= 541 | MWS +  N = 178 | P value |
| --- | --- | --- | --- | --- |
| Clinical characteristics | | | | |
| Age (years) | 57 (19) | 56 (20) | 60 (16) | **0.001** |
| Male sex *n, (%)* | 516 (72) | 380 (70) | 136 (76) | 0.125 |
| BMI (kg/m^2^) | 28 (7) | 28 (7) | 29 (8) | 0.306 |
| BSA (kg /m^2^) | 2.1 (0.4) | 2.1 (0.4) | 2.1 (0.4) | 0.244 |
| Heart rate (bpm) | 70 (20) | 70 (19) | 70 (21) | 0.6i86 |
| Systolic BP (mmHg) | 114 (22) | 115 (22) | 110 (25) | **0.025** |
| Diastolic BP (mmHg) | 69 (16) | 69 (17) | 67 (15) | 0.075 |
| Smoking (active) *n, (%)* | 138 (19) | 105 (19) | 33 (19) | 0.827 |
| Diabetes Mellitus *n, (%)* | 109 (15) | 77 (14) | 32 (18) | 0.230 |
| Hypertension *n, (%)* | 251 (35) | 181 (34) | 70 (39) | 0.174 |
| Hyperlipidemia *n, (%)* | 283 (39) | 207 (38) | 76 (43) | 0.331 |
| Atrial Fibrillation *n, (%)* | 140 (20) | 107 (20) | 33 (19) | 0.745 |
| NYHA Class III / IV *n, (%)* | 201 (28) | 140 (26) | 61 (34) | **0.034** |
| Baseline Medications | | | | |
| Beta blockers *n, (%)* | 569 (79) | 419 (78) | 150 (84) | 0.056 |
| ACEi or ARB *n, (%)* | 563 (78) | 410 (76) | 153 (86) | **0.004** |
| Entresto *n, (%)* | 59 (8) | 44 (8) | 15 (8) | 0.876 |
| Loop diuretic *n, (%)* | 197 (27) | 118 (22) | 79 (44) | **<0.001** |
| K^+^ sparing diuretic *n, (%)* | 254 (35) | 166 (31) | 88 (49) | **<0.001** |
| Thiazide diuretic *n, (%)* | 56 (8) | 44 (8) | 12 (7) | 0.630 |
| Lipid lowering (statin) *n, (%)* | 267 (37) | 195 (36) | 72 (41) | 0.325 |
| Digoxin *n, (%)* | 60 (8) | 40 (7) | 20 (11) | 0.119 |
| Anticoagulation *n, (%)* | 184 (26) | 137 (25) | 47 (26) | 0.767 |
| Anti-arrhythmic *n, (%)* | 39 (5) | 24 (4) | 15 (8) | 0.055 |
| Ca^++^ channel blocker (non-dihydropyridines) *n, (%)* | 25 (4) | 21 (4) | 4 (2) | 0.356 |
| Electrophysiology | | | | |
| PR interval (ms) | 168 (32) | 164 (36) | 172 (35) | 0.124 |
| QRS duration (ms) | 104 (48) | 104 (48) | 104 (48) | 0.493 |
| QTc (Bazzett) (ms) | 462 ± 37 | 460 +/- 38 | 465 +/- 36 | 0.149 |
| Laboratory testing | | | | |
| Hemoglobin (g/L) | 144 (23) | 143 (23) | 145 (23) | 0.150 |
| Creatinine (µmol/L) | 90 (29) | 89 (30) | 93 (31) | **0.027** |
| Sodium (mmol/L) | 140 (4) | 140 (4) | 139 (4) | 0.107 |
| CMR Imaging Characteristics | | | | |
| LVEF (%) | 40 (18) | 42 (16) | 33 (18) | **<0.001** |
| LVEF ≤35% *n, (%)* | 289 (40) | 181 (34) | 108 (61) | **<0.001** |
| LV EDV (ml) | 218 (99) | 207 (85) | 250 (112) | **<0.001** |
| LV EDVi (ml/m^2^) | 104 (43) | 101 (39) | 120 (47) | **<0.001** |
| LV ESV (ml) | 128 (84) | 120 (67) | 171 (116) | **<0.001** |
| LV ESVi (ml/m^2^) | 62 (40) | 58 (30) | 81 (49) | **<0.001** |
| LV mass (g) | 135 (63) | 130 (61) | 145 (63) | **<0.001** |
| Indexed LV mass (g/m^2^) | 66 (27) | 64 (26) | 70 (29) | **<0.001** |
| RVEF (%) | 47 (13) | 47 (12) | 47 (19) | 0.20 |
| RV EDV (ml) | 174 (77) | 172 (75) | 180 (95) | 0.107 |
| RV EDVi (ml/m^2^) | 84 (34) | 83 (31) | 87 (38) | 0.262 |
| RV ESV (ml) | 91 (59) | 90 (55) | 95 (75) | 0.102 |
| RV ESVi (ml/m^2^) | 44 (26) | 43 (24) | 46 (30) | 0.189 |
| LA vol (ml) | 81 (47) | 79 (44) | 87 (57) | **0.001** |
| Indexed LA vol (ml/m^2^) | 40 (19) | 39 (19) | 43 (24) | **0.001** |
| Any LGE (%) | 228 (32) | 50 (9) | 178 (100) | **<0.001** |
| Mid-wall striae LGE *n, (%)* | 178 (25) | 0 (0) | 178 (100) | **-** |
| Mid-wall patchy LGE *n, (%)* | 14 (2) | 13 (2) | 1 (0.6) | 0.208 |
| Sub-epicardial LGE *n, (%)* | 60 (8) | 36 (7) | 24 (14) | **0.007** |
| Diffuse LGE *n, (%)* | 3 (0.4) | 2 (0.4) | 1 (0.6) | 0.575 |

**Supplementary Table 3.**

Baseline clinical, electrocardiographic and laboratory characteristics provided for the study population, and for those with and without the **Secondary Composite Heart Failure Outcome.** Results of univariable regression analysis shown for associations with the secondary outcome.

| Variable | All subjects  N = 719 | Event -  N= 592 | Event +  N= 127 | P value | HR (95% CI)  *p<0.05 |
| --- | --- | --- | --- | --- | --- |
| Clinical characteristics | | | | | |
| Age (years) | 57 (19) | 57 (18) | 61 (19) | **0.015** | **1.02 (1.00 – 1.03)*** |
| Male sex *n* (%) | 516 (72) | 430 (73) | 86 (68) | 0.265 | 0.80 (0.55 – 1.16) |
| BMI (kg/m^2^) | 28 (7) | 28 (7) | 28 (9) | 0.182 | **1.03 (1.00 – 1.05)*** |
| BSA (kg /m^2^) | 2.1 (0.4) | 2.1 (0.4) | 2.1 (0.4) | 0.494 | 1.32 (0.74 – 2.35) |
| Heart rate (bpm) | 70 (20) | 69 (20) | 73 (21) | **0.002** | **1.02 (1.01- 1.03)*** |
| Systolic BP (mmHg) | 114 (22) | 115 (23) | 108 (26) | **0.002** | **0.98 (0.97 – 0.99)*** |
| Diastolic BP (mmHg) | 69 (16) | 70 (16) | 66 (18) | **0.039** | **0.99 (0.97 – 1.00)*** |
| Smoking (active) *n, (%)* | 138 (19) | 114 (19) | 24 (19) | 1 | 0.98 (0.63 – 1.53) |
| Diabetes Mellitus *n, (%)* | 109 (15) | 72 (12) | 37 (29) | **<0.001** | **2.72 (1.86 – 4.00)*** |
| Hypertension *n, (%)* | 251 (35) | 192 (32) | 59 (47) | **0.004** | **1.67 (1.18 – 2.37)*** |
| Hyperlipidemia *n, (%)* | 283 (39) | 222 (38) | 61 (48) | **0.035** | **1.49(1.05 – 2.11)*** |
| Atrial Fibrillation n, (%) | 140 (20) | 109 (18) | 31 (24) | 0.138 | 1.40 (0.93 – 2.10) |
| NYHA Class III / IV *n, (%)* | 201 (28) | 149 (25) | 52 (41) | **<0.001** | **2.00 (1.40 – 2.85)*** |
| Baseline Medications | | | | | |
| Beta blockers *n, (%)* | 569 (79) | 457 (77) | 112 (88) | **0.005** | **2.01 (1.17 – 3.45)*** |
| ACEi or ARB *n, (%)* | 563 (78) | 449 (76) | 114 (90) | **<0.001** | **2.59 (1.46 – 4.60)*** |
| Entresto *n, (%)* | 59 (8) | 48 (8) | 11 (9) | 0.859 | 1.17 (0.63 – 2.18) |
| Loop diuretic *n, (%)* | 197 (27) | 123 (21) | 74 (58) | **<0.001** | **4.37 (3.07 – 6.22)*** |
| K^+^ sparing diuretic *n, (%)* | 254 (35) | 192 (32) | 62 (49) | **0.001** | **1.90 (1.34 – 2.69) *** |
| Thiazide diuretic *n, (%)* | 56 (8) | 45 (8) | 11 (9) | 0.715 | 1.15 (0.617 – 2.13) |
| Lipid lowering (statin) *n, (%)* | 267 (37) | 208 (35) | 59 (47) | **0.020** | **1.54 (1.09 – 2.18)*** |
| Digoxin *n, (%)* | 60 (8) | 39 (7) | 21 (17) | **0.001** | **2.40 (1.50 – 3.84)*** |
| Anticoagulation *n, (%)* | 184 (26) | 132 (22) | 52 (41) | **<0.001** | **2.33 (1.63 – 3.32)*** |
| Anti-arrhythmic *n, (%)* | 39 (5) | 28 (5) | 11 (9) | 0.085 | 1.69 (0.91 – 3.14) |
| Ca^++^ channel blocker (non-dihydropyridines) *n, (%)* | 25 (4) | 18 (3) | 7 (6) | 0.181 | 1.75 (0.82 – 3.75) |
| Electrophysiology | | | | | |
| PR interval (ms) | 168 (32) | 164 (36) | 172 (32) | 0.154 | **1.01 (1.00 – 1.02)*** |
| QRS duration (ms) | 104 (48) | 104 (48) | 108 (48) | 0.640 | 1.00 (1.00 – 1.01) |
| QTc (Bazzett) (ms) | 462 ± 37 | 460 ± 37 | 467 ± 38 | 0.080 | 1.01 (1.00 – 1.01) |
| Laboratory testing | | | | | |
| Hemoglobin (g/L | 144 (23) | 145 (21) | 138 (28) | **0.017** | **0.99 (0.98- 1.00)*** |
| Creatinine (µmol/L) | 90 (29) | 89 (26) | 96 (39) | **0.008** | 1.00 (1.00 – 1.00) |
| Sodium (mmol/L) | 140 (4) | 140 (3) | 139 (4) | **0.020** | **0.92 (0.87 – 0.97)*** |
| CMR Imaging characteristics | | | | | |
| LVEF (%) | 40 (18) | 42 (17) | 34 (20) | **<0.001** | **0.97 (0.95 – 0.98)*** |
| LVEF ≤35% *n, (%)* | 289 (40) | 214 (36) | 75 (59) | **<0.001** | **2.40 (1.69 – 3.43)*** |
| LV EDV (ml) | 218 (99) | 213 (90) | 242 (126) | **0.008** | **1.00 (1.00 – 1.01)*** |
| LV EDVi (ml/m^2^) | 104 (43) | 103 (41) | 113 (60) | **0.013** | **1.01 (1.00- 1.01)*** |
| LV ESV (ml) | 128 (84) | 125 (73) | 162 (128) | **<0.001** | **1.00 (1.00 – 1.01)*** |
| LV ESVi (ml/m^2^) | 62 (40) | 60 (35) | 74 (64) | **<0.001** | **1.01 (1.00 – 1.01)*** |
| LV mass (g) | 135 (63) | 132 (59) | 153 (71) | **<0.001** | **1.01 (1.00- 1.01)*** |
| Indexed LV mass (g/m^2^) | 66 (27) | 65 (25) | 73 (35) | **<0.001** | **1.02 (1.01 – 1.02)*** |
| RVEF (%) | 47 (13) | 48 (13) | 43 (16) | **0.001** | **0.98 (0.96 – 0.99)*** |
| RV EDV (ml) | 174 (77) | 175 (76) | 165 (87) | 0.613 | 1.00 (1.00 – 1.00) |
| RV EDVi (ml/m^2^) | 84 (34) | 85 (33) | 82 (38) | 0.266 | 1.00 (0.99- 1.01) |
| RV ESV (ml) | 91 (59) | 89 (56) | 97 (71) | 0.249 | 1.00 (1.00 – 1.01) |
| RV ESVi (ml/m^2^) | 44 (26) | 43 (24) | 47 (28) | 0.309 | 1.01 (1.00 – 1.02) |
| LA vol (ml) | 81 (47) | 80 (43) | 91 (57) | **0.002** | **1.01 (1.00 – 1.01)*** |
| Indexed LA vol (ml/m^2^) | 40 (19) | 39 (19) | 43 (29) | **0.003** | **1.02 (1.01 – 1.02) *** |
| Any LGE (%) | 228 (32) | 172 (29) | 56 (44) | **0.002** | **1.76 (1.24– 2.50)*** |
| Mid-wall striae LGE *n, (%)* | 178 (25) | 127 (22) | 51 (40) | **<0.001** | **2.15 (1.50 – 3.06)*** |
| Mid-wall patchy LGE *n, (%)* | 14 (2) | 12 (2) | 2 (2) | 1 | 0.76 (0.19 – 3.09) |
| Sub-epicardial LGE *n, (%)* | 60 (8) | 50 (9) | 10 (8) | 1 | 0.95 (0.50– 1.82) |
| Diffuse LGE *n, (%)* | 3 (0.4) | 2 (0.4) | 1 (0.8) | 0.442 | 1.74 (0.24 – 12.4) |

**Supplementary Table 4.**

Baseline clinical, electrocardiographic and laboratory characteristics provided for the study population, and for those with and without the **Secondary Composite Arrhythmia Outcome.** Results of univariable regression analysis shown for associations with the secondary outcome.

| Variable | All subjects  N = 719 | Event -  N= 674 | Event +  N= 45 | P value | HR (95% CI)  *p<0.05 |
| --- | --- | --- | --- | --- | --- |
| Clinical characteristics | | | | | |
| Age (years) | 57 (19) | 55 (14) | 56 (16) | **0.92** | 1.00 (0.98 – 1.02) |
| Male sex *n* (%) | 516 (72) | 478 (71) | 38 (84) | 0.06 | 2.15 (0.96 – 4.81) |
| BMI (kg/m^2^) | 28 (7) | 29 (6) | 32 (10) | **0.03** | **1.07 (1.03 – 1.11)*** |
| BSA (kg /m^2^) | 2.1 (0.4) | 2.1 (0.30) | 2.2 (0.38) | **0.003** | **3.97 (1.62 – 9.74)*** |
| Heart rate (bpm) | 70 (20) | 71 (16) | 70 (16) | 0.71 | 1 (0.98 – 1.02) |
| Systolic BP (mmHg) | 114 (22) | 116 (18) | 115 (16) | 0.75 | 1 (0.98 – 1.01) |
| Diastolic BP (mmHg) | 69 (16) | 70 (13) | 67 (10) | 0.08 | 0.98 (0.96 – 1.01) |
| Smoking (active) *n, (%)* | 138 (19) | 126 (19) | 12 (27) | 0.24 | 1.63 (0.84 – 3.15) |
| Diabetes Mellitus *n, (%)* | 109 (15) | 97 (14) | 12 (27) | **0.03** | **2.15 (1.11 – 4.17)*** |
| Hypertension *n, (%)* | 251 (35) | 229 (34) | 22 (49) | **0.05** | **1.80 (1- 3.21)*** |
| Hyperlipidemia *n, (%)* | 283 (39) | 259 (38) | 24 (53) | 0.06 | 1.77 (0.99 – 3.18) |
| Atrial Fibrillation n, (%) | 140 (20) | 130 (19) | 10 (22) | 0.70 | 1.19 (0.60 – 2.40) |
| NYHA Class III / IV *n, (%)* | 201 (28) | 184 (27) | 17 (38) | 0.168 | 1.65 (0.90 – 3.01) |
|  | | | | | |
| Beta blockers *n, (%)* | 569 (79) | 532 (79) | 37 (82) | 0.71 | 1.16 (0.54 – 2.50) |
| ACEi or ARB *n, (%)* | 563 (78) | 527 (78) | 36 (80) | 0.85 | 1.10 (0.53 – 2.28) |
| Entresto *n, (%)* | 59 (8) | 53 (8) | 6 (13) | 0.253 | 1.85 (0.78 – 4.38) |
| Loop diuretic *n, (%)* | 197 (27) | 176 (26) | 21 (47) | **0.005** | **2.40 (1.34 – 4.31)*** |
| K^+^ sparing diuretic *n, (%)* | 254 (35) | 238 (35) | 16 (36) | 1 | 1.02 (0.55 – 1.88) |
| Thiazide diuretic *n, (%)* | 56 (8) | 52 (8) | 4 (9) | 0.77 | 1.19 (0.43 – 3.32) |
| Lipid lowering (statin) *n, (%)* | 267 (37) | 244 (36) | 23 (51) | 0.06 | 1.78 (0.99 – 3.20) |
| Digoxin *n, (%)* | 60 (8) | 57 (9) | 3 (7) | 1 | 0.74 (0.23- 2.39) |
| Anticoagulation *n, (%)* | 184 (26) | 170 (25) | 14 (31) | **0.38** | 1.34 (0.71 – 2.52) |
| Anti-arrhythmic *n, (%)* | 39 (5) | 31 (5) | 8 (18) | 0.02 | **4.16 (1.94 – 8.94)*** |
| Ca^++^ channel blocker (non-dihydropyridines) *n, (%)* | 25 (4) | 23 (3) | 2 (4) | 0.67 | 1.33 (0.32 – 5.49) |
|  | | | | | |
| PR interval (ms) | 168 (32) | 169 (29) | 175 (26) | 0.27 | 1.01 (1 – 1.02) |
| QRS duration (ms) | 104 (48) | 115 (31) | 123 (30) | 0.14 | 1.01 (1 – 1.02) |
| QTc (Bazzett) (ms) | 462 ± 37 | 461 (37) | 466 (41) | 0.45 | 1.00 (1 – 1.01) |
|  | | | | | |
| Hemoglobin (g/L | 144 (23) | 142 (17) | 142 (17) | 0.93 | 1 (0.98- 1.02) |
| Creatinine (µmol/L) | 90 (29) | 97 (66) | 97 (32) | 0.93 | 1 (1- 1.01) |
| Sodium (mmol/L) | 140 (4) | 139 (3) | 138 (3) | 0.06 | 0.91 (0.83 – 1) |
|  | | | | | |
| LVEF (%) | 40 (18) | 37 (11) | 35 (10) | 0.12 | 0.98 (0.96 – 1.01) |
| LVEF ≤35% *n, (%)* | 289 (40) | 264 (39) | 25 (56) | **0.04** | **1.93 (1.07 – 3.48)*** |
| LV EDV (ml) | 218 (99) | 230 (83) | 275 (102) | **<0.001** | **1.01 (1 – 1.01)*** |
| LV EDVi (ml/m^2^) | 104 (43) | 112 (39) | 125 (41) | **0.03** | **1.01 (1- 1.01)*** |
| LV ESV (ml) | 128 (84) | 150 (77) | 186 (91) | **0.02** | **1 (1- 1.01)*** |
| LV ESVi (ml/m^2^) | 62 (40) | 73 (37) | 85 (40) | **0.04** | **1.01 (1 – 1.01)*** |
| LV mass (g) | 135 (63) | 142 (50) | 172 (63) | **0.003** | **1.01 (1 – 1.01)*** |
| Indexed LV mass (g/m^2^) | 66 (27) | 69 (22) | 78 (26) | **0.007** | **1.02 (1– 1.03) *** |
| RVEF (%) | 47 (13) | 46 (12) | 44 (11) | **0.27** | 0.99 (0.96 – 1.01) |
| RV EDV (ml) | 174 (77) | 181 (61) | 189 (61) | 0.38 | 1 (1 – 1.01) |
| RV EDVi (ml/m^2^) | 84 (34) | 87 (25) | 86 (23) | 0.68 | 1 (0.99 – 1.01) |
| RV ESV (ml) | 91 (59) | 101 (50) | 107 (44) | 0.41 | 1 (1 – 1.01) |
| RV ESVi (ml/m^2^) | 44 (26) | 49 (22) | 49 (19) | 0.95 | 1 (0.99 – 1.01) |
| LA vol (ml) | 81 (47) | 89 (40) | 90 (39) | 0.91 | 1 (0.99 – 1.01) |
| Indexed LA vol (ml/m^2^) | 40 (19) | 43 (18) | 41 (16) | 0.43 | 0.99 (0.98 – 1.01) |
| Any LGE (%) | 228 (32) | 206 (31) | 22 (49) | **0.01** | **2.14 (1.19 – 3.84)*** |
| Mid-wall striae LGE *n, (%)* | 178 (25) | 159 (24) | 19 (42) | **0.007** | **2.31 (1.28 – 4.17)*** |
| Mid-wall patchy LGE *n, (%)* | 14 (2) | 12 (2) | 1 (2) | 0.60 | 1.11 (0.15. – 8.09) |
| Sub-epicardial LGE *n, (%)* | 60 (8) | 56 (8) | 4 (9) | 0.783 | 1.1 (0.39 – 3.07) |
| Diffuse LGE *n, (%)* | 3 (0.4) | 2 (0.3) | 1 (2) | 0.18 | 6.27 (0.86 – 45.7) |
